# Developments of Smart Drug-Delivery Systems Based on Magnetic Molecularly Imprinted Polymers for Targeted Cancer Therapy: A Short Review

**DOI:** 10.3390/pharmaceutics12090831

**Published:** 2020-08-31

**Authors:** Nasim Sanadgol, Judith Wackerlig

**Affiliations:** Department of Pharmaceutical Chemistry, Faculty of Life Sciences, University of Vienna, 1090 Vienna, Austria; a11742860@unet.univie.ac.at

**Keywords:** molecular imprinting technology, magnetic molecularly imprinted polymers, magnetic nanoparticles, chemotherapy, cancer, smart-drug-delivery system

## Abstract

Cancer therapy is still a huge challenge, as especially chemotherapy shows several drawbacks like low specificity to tumor cells, rapid elimination of drugs, high toxicity and lack of aqueous solubility. The combination of molecular imprinting technology with magnetic nanoparticles provides a new class of smart hybrids, i.e., magnetic molecularly imprinted polymers (MMIPs) to overcome limitations in current cancer therapy. The application of these complexes is gaining more interest in therapy, due to their favorable properties, namely, the ability to be guided and to generate slight hyperthermia with an appropriate external magnetic field, alongside the high selectivity and loading capacity of imprinted polymers toward a template molecule. In cancer therapy, using the MMIPs as smart-drug-delivery robots can be a promising alternative to conventional direct administered chemotherapy, aiming to enhance drug accumulation/penetration into the tumors while fewer side effects on the other organs. Overview: In this review, we state the necessity of further studies to translate the anticancer drug-delivery systems into clinical applications with high efficiency. This work relates to the latest state of MMIPs as smart-drug-delivery systems aiming to be used in chemotherapy. The application of computational modeling toward selecting the optimum imprinting interaction partners is stated. The preparation methods employed in these works are summarized and their attainment in drug-loading capacity, release behavior and cytotoxicity toward cancer cells in the manner of in vitro and in vivo studies are stated. As an essential issue toward the development of a body-friendly system, the biocompatibility and toxicity of the developed drug-delivery systems are discussed. We conclude with the promising perspectives in this emerging field. Areas covered: Last ten years of publications (till June 2020) in magnetic molecularly imprinted polymeric nanoparticles for application as smart-drug-delivery systems in chemotherapy.

## 1. Introduction

### 1.1. Hurdles in Chemotherapy

Cancer is one of the most difficult-to-manage diseases, causing a vast amount of mortality around the world, with more than 10 million new patients each year. The three main approaches in cancer therapy are surgical removal, irradiation and chemotherapy. The cancer type and development stage determine the comparative value of these approaches. However, the most applied strategy implemented for localized and metastatic cancer treatment is chemotherapy, which is carried out alone or combined with other treatment approaches [1]. Conventional direct administration of chemotherapeutic agents shows several serious hurdles, including low or no specificity to tumor cells and consequently low discrimination between cancer cells and healthy cells, rapid elimination of drugs from the body, substantial multidrug resistance, lack of aqueous solubility, poor oral bioavailability, narrow therapeutic windows, restricted cellular penetration and low therapeutic indices [1,2,3]. The direct systemic administration leads to extreme fluctuation in the drug plasmatic concentration causing high toxicity, poor specificity and massive side effects on healthy cells. These drawbacks associated with the conventional chemotherapeutic agents as the leading causes of the dramatic decrease of their therapeutic value should be addressed with novel strategies [4], concerning the use of tumor-targeted delivery systems capable of promoting specific drug accumulation at the pathologic site.

Unfortunately, these drastic adverse effects enforced by chemotherapeutic agents on healthy organs are one of the main reasons for the vast mortality rate of cancer patients. On the other hand, the relatively weak bio-accessibility and penetration of these drugs to tumor tissues show the need for higher doses leading to increased toxicity and the incidence of multiple drug resistance [5]. Multidrug resistance (MDR) is one of the fundamental obstacles for several chemotherapeutic agents like 5-fluorouracil (5-FU) to effect efficiently in therapy [6]. Efficiency in drug delivery means the safe transport of the drug to target sites without significant degradation of the drug and harming the body [7]. An ideal drug-delivery system (DDS) can ensure the release of the therapeutic agent at the right site and in the right dosage for the required period to maximize its efficacy by the accumulation at the site of action and reach the therapeutic concentration level within the therapeutic window while minimizing the side effects on healthy tissues [3,8]. Furthermore, this delivery system necessarily should be biocompatible and biodegradable to be able to enter the body without specific toxicity, immunogenicity and accumulation in other organs than the tumor [4].

Keeping the main focus on the anticancer drugs, in the next subsections, we first give a brief overview of the developed systems as DDS, their features and obstacles, followed by a description of the development of DDSs based on the recently developed molecularly imprinted multifunctional polymers (MIPs) alone as well as combined with highly interested magnetic materials. Section 2 highlights the latest studies of this versatile combination as a targeted DDS in the delivery of chemotherapy agents to the tumor location and enhancement of their therapeutic efficacy, stating what currently are the computational pre-screening methods, preparation methods and the in vitro/vivo outcomes from different aspects with an emphasize on safety regulation of the obtained systems. We conclude with an outlook on the role of these systems in anticancer drug-delivery technology, suggesting further study aspects.

### 1.2. Nano-Size Delivery Systems

Over the years, numerous studies have been performed to develop nanosize DDSs with the broad range of different materials and anticancer drugs, alongside with the nanotechnology that has emerged as a powerful tool for drug delivery. Altogether showing a massive potential in terms of pharmacological enhancement and control over drugs performance in chemotherapy [9,10]. The use of nanotechnology for cancer treatment is an active area of biomedical research [7]. Nanomaterials had a strong influence on developing promising DDSs [11]. The wide range of materials including natural polymers (biopolymers) [12,13,14], (semi) synthetic polymers [13,15] in forms of polymeric nanoparticles (NPs), micelles, vesicles or dendrimers [16], as well as lipids (liposomes) [17] and inorganic materials [13], have been employed to develop drug-delivery complexes with high biologic efficacy.

Liposomal nanodelivery of chemotherapeutics as the first generation of nanosize DDSs indeed became the most successful DDS in chemotherapy by the number of encapsulated anticancer drugs such as daunorubicin, vincristine, irinotecan and doxorubicin that have entered several stages of clinical trials [18] and several formulations approved by the US Food and Drug Administration (FDA) such as Myocet^®^, Daunoxome^®^, Doxil^®^/Caelyx^®^ and the most recently approved liposomal DDS, Onivyde^®^ (liposomal irinotecan), approved as a second-line treatment for metastatic pancreatic cancer [19,20,21]. However, among considerable shortcomings of liposomes are the low capacity to encapsulate lipophilic drugs, manufacture processes involving organic solvents, often leakage and instability in biologic fluids and aqueous solutions [22,23].

Polymeric NPs are also extensively employed as biomaterials because of their favorable characteristics. About 30% of total nanomedicines approved by the FDA from the mid-1990 s to 2016 belong to polymeric NPs, due to high synthetic versatility and ease of modifications [24], showing much reduced adverse effects compared to bare drugs. Copaxone^®^ (a random copolymer composed of l-glutamic acid, l-alanine, l-lysine and l-tyrosine [25] used in multiple sclerosis) and Neulasta^®^ (PEGylated GCSF protein for the treatment of neutropenia in chemotherapy), are two polymeric NP formulations, which ranked among the top 10 best-selling drugs in the US in 2013 [26,27] as well as the FDA approved polymeric NP for cancer therapy, Eligard^®^ (Tolmar) (Leuprolide acetate and polymer (PLGH (poly (dl-lactide-*co*-glycolide) in prostate cancer) [21].

All these DDSs based on polymeric NPs were introduced to decrease traditional drug administration challenges but come with their obstacles and constraints, emphasizing the importance of further research [21,28]. There is no consensus about the actual therapeutic efficacy of the developed NPs toward cancer therapy because of many different kinds of NP treatment techniques that are used. It can be said that, despite encouraging remarkable results with polymeric NPs toward cancer therapy, there has been limited clinical advancement [7,29]. It is hard to conclude whether they are equal to or better than conventional treatments in regards to “treating” cancer [7]. Lack of therapeutic-acceptable drug-loading capacity and initial fast premature drug release leads to a suboptimal activity at the targeted site [4]. Dose-dumping induced toxicity, inconsistent release pattern [2], changes in the physicochemical properties of the NPs in the systemic circulation such as in particle size and aggregation behavior [28] are some of the persisted limits with these formulations and arise the necessity of further studies to address these issues. As analyzed by Wilhelm et al. [30] in the literature from the past ten years on NP-based drug carriers, only 0.7% of the administered NP dose was delivered to a solid tumor. This low delivery efficiency negatively affects the translation of nanotechnology to clinical applications [10]. Most of these systems reach the site of action passively using the enhanced permeability retention effect (EPR) offered by the vascular permeability and lack of lymphatic drainage around tumors that facilitate the extravasation and accumulation of NPs passively within cancer cells [31]. Hence, many research groups focus on active tumor-targeting by optimization, surface modification and drug-triggered-release of NPs to escape immune clearance, avoid nonspecific cell uptake, stick to the target tissues and interact by desired cells [22,32,33] that could address the tumor tissue directly and enhance chemotherapeutic efficacy [34]. However, despite the significant findings and potential to impact drug clinical features, only marginal progress has achieved in their therapeutic efficacies toward their clinical application. Therefore, the current focus on developing nanomedicines of high therapeutic index lies in tailoring the fundamental physicochemical properties of NPs, most importantly, selectivity, stability, surface properties and size [22]. Among various DDSs, one of the newly viable developed strategies for this aim could be molecular imprinting technology (MIT) to generate new nanoscale and larger tailor-made pharmacological complexes [4].

### 1.3. Molecularly Imprinted Technology toward Drug-Delivery System (DDS)

**Figure 1:**
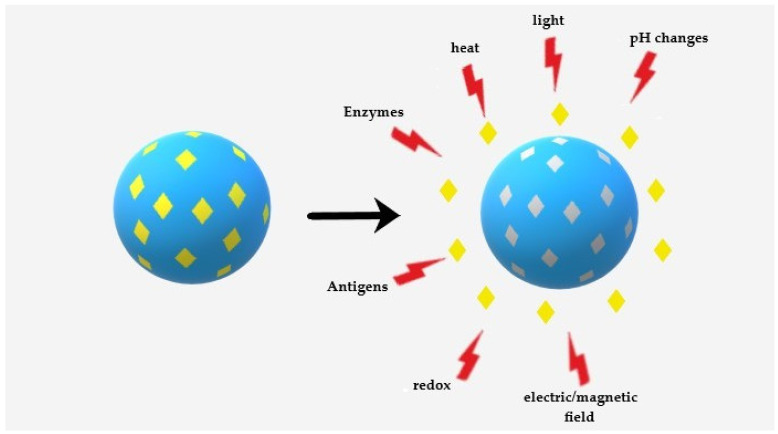
Stimuli-sensitive release of the cargo upon applying specific release triggers. The polymeric network can be designed to become remotely controllable through sensitivity to such external stimuli.

MIT is a step further into the design of polymeric NPs. This technology has become an established strategy, but it is still considered a burgeoning method toward biomedical applications. MIT allows for producing smart materials in nano and larger sizes with active sites that match the target compound’s size and functionality, the so-called template, within a polymeric matrix. Generally, the copolymerization of a liquid mixture containing porogenic solvent(s), functional monomers, template molecules and crosslinkers with a careful design leads to the development of molecularly imprinted polymers (MIPs). The responsibility of creating intermolecular interactions with the template molecule is by functional monomers through either covalent or non-covalent bonds, whereas crosslinkers form the polymer scaffold around the template [35]. The obtained MIPs possess tailored cavities resembling the original template in terms of size, shape and orientation [36]. Regarding drug delivery, due to the intermolecular interactions like hydrogen bonds, dipole-dipole and ionic interactions between the template molecule and polymer functional groups, these cavities are capable of enhancing the NPs loading capacity, improving drug stability, solubility and adjusting the drug release kinetics [4,37,38]. An intelligent or smart drug release is the anticipated release of a therapeutic agent on-demand. For this aim, these MIPs can react to the external stimulations, making changes in their structure or the strength of interactions between the polymer functional groups and the template captured in the cavities. This feature is highly suitable for DDS as it allows the drugs to be released only upon a particular change in the environment (Figure 1) [35] such as heat, pH changes, light, electric or magnetic fields, enzymes, reduction and ultrasound waves [8,29]. The combination of stimuli-sensitivity and imprinting technology potentially leads to a high loading capacity of the template by imprinting, while the response to the external stimuli modulates the affinity of the polymeric network for the template molecule, providing the regulatory or switching capability of the loading/release processes [37].

The main advantages of MIPs in comparison with biomolecules such as antibodies and biologic receptors are their relatively high stability over various conditions and low cost [37]. MIPs have stable spatial structure and long-lasting shelf life that can be up to several years at room temperature [38] and exceptional physical robustness and stability against tough conditions, including highly acidic and basic pH, temperature fluctuation, organic solvents and mechanical and thermal pressures [36,39,40]. In addition, compared to the non-imprinted polymeric NPs, the chief advantages of MIPs are their high selectivity and affinity for the target molecule, leading to the higher loading capacity and potential lower dose-dumping and immature burst release of the cargo [38]. They have been implemented in the development of biosensors [41], antibody mimics [42], catalysis [43], molecular recognition [44], drug delivery [45,46], diagnostics [47] and other biomedical applications [36]. As drug-delivery carriers, they have favorable specific binding tendency and loading, stability under different harsh settings, flexibility and antibody-like recognition [48].

Noticeable progress has been gained in this area and many nanoMIP-based DDSs with largely improved sustained release drug-delivery ability compared to their control polymers have been developed for different kinds of drugs intended to be used in various diseases [49]. The constraints concerning MIPs still need to be addressed, such as slow binding kinetics, aqueous compatibility, permeability in order to the drug extraction and heterogeneity of binding site distributions [8,37]. However, there is a high chance for anticancer drugs to be transported by these carriers easily cross the cytoplasmic and nuclear membrane that will bring them with the in situ delivery with intact concentration and consequently, higher efficiency in the elimination of the tumor cell than that administered alone by conventional chemotherapy [50].

Anticancer drugs such as doxorubicin, 5-fluorouracil and paclitaxel were utilized as a template of MIPs often to achieve controlled/sustained release of these drugs as well as better bioavailability, protection of the drug from fast degradation, diminish the adverse effects and efficient localized effect for potential chemotherapy of various cancers [51,52,53,54,55]. Bai et al. reported high drug loading (17.1%) and encapsulation efficiency (85.5%), as well as the desirable pH-dependent release (much faster release at pH 5 than those at pH 7) with a very slow and controlled release of paclitaxel imprinted system [51]. Similar outcomes were also reported by other groups [52,54].

The concept of MIT has a long history back to the early 1930 s. However, the preparation of organic polymers with molecular recognition as we know it today was first reported only in 1972 when two independent laboratories of Wulff and Klotz reported the preparation of organic polymers with a preselected ligand. Template molecules that were present during polymerization or derivatives were recognized better by the resultant structures [37,56,57]. Later on, a magnetically assisted DDS (MADDS) was introduced by Widder and coworkers in 1978, applying inorganic magnetic material in the structure of MIPs [58]. The combination of a magnetic core covered by a thin MIP shell leads to the generation of smart hybrid structures, namely magnetic MIPs (MMIPs) that provides the possibility of high drug loading and low off-target drug release followed by remote guidance, rapid distribution and local accumulation of the obtained MMIPs by using an external magnetic field [59]. Due to the good biocompatibility and chemical, thermal and mechanical stability, high sorption capacity, high selectivity, reusability, low cost and facile preparation method of the available magnetic materials, especially magnetic NPs (MNPs), the design of MMIPs as DDSs is recently become favorable [60,61]. Due to the high surface-to-volume-ratio of MNP, compared to MIPs, imprinting position of the polymer at the surface is increased leading to MMIPs with more accessible imprinted positions, rapid mass transfer and hence, fewer permeability issues, as well as strong anti-interference ability [62].

One of the main obstacles toward nanocarriers’ efficacy in drug delivery could be the lack of knowledge about the precise bio-distribution, location and subsequent therapeutic effects, as most studies have not examined the targeting efficiency of NPs real time in vivo [28]. With this regard, among the active targeted MIP-based systems [53,54,63,64,65,66], MMIPs are appealing [53,64,65] because of the ease of active remote guidance to the site of interest in the body by using an external magnetic field. Regarding tumor chemotherapy, this feature largely enhances the drug concentration in the tumor tissues by much lower costs and potentially improves its therapeutic efficacy while narrowing the adverse toxicity to healthy cells through tumor local accumulation [49].

### 1.4. Magnetic Molecularly Imprinted Polymers, Promising Hybrid Nano-DDS

Besides ligand-mediated targeting, physical targeting can be achieved by adding some specific physical properties to the DDSs. One of the most interesting features could be the magnetic force which can accumulate magnetic materials in the specific region, in this content, tumor location, by use of a magnetic field [22].

Nowadays, MNPs, find vast applications in medicine, analytical chemistry and biotechnology. The most commonly used MNPs includes metal or metal oxide NPs [67]. Iron oxide-based MNPs (Fe_2_O_3_, Fe_3_O_4_) especially the only clinically approved MNP, superparamagnetic iron oxide NP (SPION) are extensively investigated in nanomedicine for their biocompatibility, stability, eco-friendliness, low toxicity, contrast agent properties, ability to generate heat when submitted to an alternating magnetic field (hyperthermia) and intrinsic magnetic properties, i.e., superparamagnetism that allows them to exhibit magnetic properties only in the presence of an applied magnetic field [9,68]. Considering the property of superparamagnetism, they are broadly investigated in different clinical applications [67], especially as imaging agents. Feridex^®^/Endorem^®^ and GastroMARK™; Umirem^®^ (AMAG pharmaceuticals) are SPION NPs coated with dextran and silicone, respectively that due to their superparamagnetic character, were approved as imaging agents [21].

Magnetic drug targeting (MDT) involves enriching SPIONs at the area of interest via a strong external magnetic field and, consequently, potentially enables more specific and efficient treatment. MDT of drug-loaded SPIONs is indeed closer to application in patients [22]. Successful employment of SPIONs for cancer treatment was demonstrated by the complete tumor remission without significant side effects followed by the administration of mitoxantone−SPIONs (with only 5–10% of the conventional chemotherapeutic dose) through the tumor-supplying vessel in rabbits and the application of a strong external magnetic field over the tumor location. The distribution profile after MDT displayed 66.3% of the particles localized in the tumor region with magnetic targeting, compared to less than 1% of drug and NPs reaching the tumor region during conventional intravenous application [69].

Furthermore, applying a thin imprinted polymer shell on the surface of the MNPs leads to enhancement in physicochemical properties for the intended MDT by enhancing binding kinetic, high surface-to-volume-ratio, increasing binding capacity, uniform spherical shape and also monodispersity in aqueous blood circulatory [62]. Due to the presence of MNPs in their structure, they can induce the so-called magnetic hyperthermia (local heat enhancement) when submitted to an external magnetic field (subsequent release of the drug when MMIP is loaded). This feature is exclusively efficient for the demolition of cancer cells, which cannot survive in the temperature range of 40–48 °C, unlike healthy cells that can endure such temperatures with insignificant or no injury [9,70,71]. It is well known for over three decades that tumor cells have a significant sensitivity to moderate hyperthermia in “fever-range” temperatures (41–45 °C) than normal cells, as usually, the consequences of hyperthermia on healthy cells show up at temperatures >50 °C with coagulation [71]. Nanotherm™ (MagForce) consists of amino silane-coated SPIONs and designed for tumor therapy (glioblastoma) using local tissue hyperthermia [72,73]. Nanotherm™ is already marketed in Europe for the thermotherapy of glioblastoma and is in late-stage clinical trials in the US, and FDA approval is pending [74,75]. Here, the magnetic fluid is injected directly into the tumor and then an alternating magnetic field applicator which changes its polarity up to 100,000 times per second is used to selectively heat the particles, resulting in local heating of the tumor environment (temperatures reach 40–45 °C), leading to cell death [72] and increase in the overall survival of up to 12 months [76].

## 2. State of the Art

In the last decade, the preparation and application of different types of magnetic nanocarriers for the delivery of chemotherapy agents were well studied and fairly reviewed [9]. However, the employment of MMIPs as carriers of anticancer agents is emerging. Only a few publications reported the development of MMIPs with the aim of smart delivery of anticancer drugs local to the tumor [3,50,54,61,65,68,77,78,79,80,81] that shed light on the development of a novel generation of multifunctional hybrid DDSs and future perspectives in this field.

**Figure 2:**
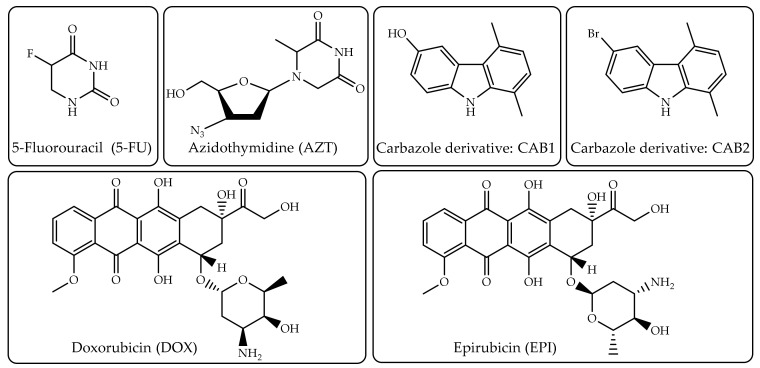
Structures of anticancer drugs applied for drug delivery by magnetic molecularly imprinted polymers (MMIPs) systems discussed in this review and published so far.

5-fluorouracil (5-FU), doxorubicin (DOX), carbazole derivatives (CAB1, CAB2), epirubicin (EPI) and azidothymidine (AZT) were chosen as anticancer drug templates for this purpose (Figure 2). The development methods employed are summarized, and their achievements so far on in vitro and in vivo studies are stated in the following.

5-FU, DOX and EPI with a broad antineoplastic spectrum are used to treat many different types of cancer as colon or rectum, breast, liver, bladder and brain [50,61,79,80]. The main drawbacks of direct chemotherapy with these agents can be listed as severe depression of hematopoiesis (anemia, leucopenia, thrombopenia), infection and cardiotoxicity regarding EPI [50], 5-FU resistance and rapid clearance from the body [6] and DOX cumulative cardiotoxicity and nephrotoxicity [82]. Overall, these severe side effects had a deep impact on the mortality rate of cancer patients [83].

## 3. Magnetic Molecularly Imprinted Polymer (MMIP) Preparation Methods

The development of MMIPs usually includes synthesis of MNPs followed by the surface protection and functionalization of these MNPs, template attachment and decoration of pre-polymerization complex with monomers and crosslinker(s) and polymerization. Template removal is performed afterwards to achieve the empty imprinted cavities for analytical applications such as recognition and isolation [59]. In the case of DDS, the last step may not be necessary. The interaction of the functional monomer and the template in this system is governed by equilibrium. The functional monomers normally must be added in excess, relative to the number of moles of the template to favor the formation of the complex leading to the several configurations of the template-functional monomer complex with a range of affinity constants. The crosslinker keeps control over the morphology of the polymer matrix, serves to stabilize the imprinted binding sites and gives mechanical stability to the polymer matrix to retain its molecular selectivity via imprinted cavities [38]. Ethylene glycol dimethacrylate (EGDMA) and trimethylolpropane trimethacrylate (TRIM) are the most common crosslinkers and have an impact on the physical characteristics of the polymers and show a low effect on the specific interactions between the template and functional monomers [4,38]. TRIM, as a crosslinker, gives polymers with more rigidity, structure order and effective binding sites than EGDMA [38]. It is necessary to note that in all the following studies, the magnetic non-imprinted polymers (MNIPs) are also prepared by the same synthesis procedure without adding the template molecule in the preparation steps.

The preparation of magnetic Fe_3_O_4_ NPs is commonly performed by a chemical co-precipitation of Fe^2+^ and Fe^3+^ ions of FeCl_2_∙4H_2_O and FeCl_3_∙6H_2_O at 80 °C in the presence of ammonia (sodium hydroxide solution, 2.0 mol·L^−1^) with high yield and high sorption capacity of the resulting particles [67,84]. These obtained MNPs can aggregate and degrade gradually by oxidation, which reduces their magnetization capacity [84]. Therefore, their long-term stability and surface functionalization are fundamental issues concerning these iron-based MNPs [85]. In addition, a hydrophobic surface with a large surface-area-to-volume ratio is unfavorable to be implemented in any biologic accepted fluids [59,86]. Hence, for biomedical applications, it is desirable to modify MNPs with a surface coating layer for their stability in suspension, protection against oxidation and in vivo biocompatibility [84].

There are several well-developed methods for this purpose that can be divided into two main groups, organic and inorganic coating. Among inorganic coating materials, silica and gold (Au) are frequently used as MNP coating material. Dextran, alginate, starch, chitosan, silanes, glycosaminoglycan, sulfonated styrene-divinylbenzene, polyethylene glycol (PEG), polyvinyl alcohol, poly (methyl methacrylate), polyacrylic acid and dendrimer shell are broadly used organic coatings materials of MNPs [59]. The choice of the coating material highly depends on the other interaction partners in the imprinting process as well as the final application of the designed system as each of them offers different properties to the system. However, coating with materials with functional groups offers the possibility to immobilize other materials at the surface prior to the imprinting process, leading to a stable pre-polymerization complex and higher imprinting efficiency, respectively. Polymerizable vinyl groups offered by silane coupling agents are good examples and widely used as this feature is highly directed to the selective occurrence of molecular imprinting polymerization at the surface of MNPs [59,61,87].

The Griffete group utilized this feature in their imprinting process to obtain DOX-loaded MMIPs for drug delivery. They performed the functionalization of MNPs by merely growing a thin polymer layer of acrylic acid (AA) monomers on their surface, forming a molecule monolayer with polymerizable vinyl end groups [68,77]. The process is a simple complexing reaction of AA with unsaturated iron ions of the MNP surface. Subsequently, in the presence of functional monomers and crosslinkers, the AA monolayer will direct the selective occurrence of polymerization at the surface of MNPs [68,77,87]. This simple approach was rapidly used in other studies as a bullet point to increase drug loading and imprinting yield [78,88].

### 3.1. Choose of Suitable Monomers with the Aid of Computational Modeling

As it is well shown in most studies on MIT, limitations occur in the preparation of imprinted systems with the choice of the bond type and monomers used to attach the drug template with these designed systems. The optimal template- monomer- crosslinker- solvent interactions strongly affect the successful imprinting process [89].

The selection of the best functional monomers is usually made using different formulations with various monomers or in the manner of a trial-and-error procedure that is time-consuming and expensive [89,90]. Nowadays, the use of computational simulation is suggested toward the rational design of imprinting systems for the optimization of monomer preselection based on potential monomer-template complex conformation by the comparison of the binding energy between the template and functional monomers and the utilizing of molecular docking platforms to predict several modes of interactions between the reaction counterparts [91]. Indeed, molecular modeling and computational approach could facilitate the MIP development [92]. Wu et al. [93] were one of the pioneers of employing a computational approach to study the nature of recognition of MIPs. They reported a correlation between the binding energy of the template and functional monomer and the capacity factor via the production of high-affinity binding sites in the obtained polymer. There are several computational chemistry approaches based on molecular mechanics (MM) and molecular dynamics (MD) aiming to perform predictive analyses of such complexes intermolecular as well as in interaction with their environment, utilizing quantum methods (like ab initio mechanical quantum calculations and semi-empirical methods) [89,91,93], solubility parameters [94] and geometric parameters [89]. Several groups developed strategies for the rational design of MIPs through molecular modeling [40,50,61,80,89,95,96,97,98,99]. Nevertheless, there is no consensus on the most effective computational model in the prediction and determination of the properties of the designed MIP [90]. These approaches are implemented in several widely used software packages under different methods [90,100], and the need for a comprehensive classification and application of these methods in the rational design of imprinted polymeric nanocarriers greatly exists. We briefly introduce the methods and parameters implemented by the studies of interest of this review in the following.

#### 3.1.1. Cohesion Parameters

The first insight into the computational modeling of the intended complex can be the investigation of the solubility and miscibility of the components. The compatibility and miscibility of functional monomers can be determined prior to the experimental step by measuring solubility parameters, as well as the cohesive energy density of components (CED) [61]. With this regard, Talavat and Güner chose optimal monomers to synthesize pH-responsive 5-FU-loaded MMIPs based on the chemical affinity profiles of the Hansen method, a thermodynamic computational calculation method, using the “Hansen solubility parameter calculation program” (HSPCP) containing data for the polymers and solvents [61]. The Hansen solubility parameter (δ) includes δd, δp and δH subparameters, which δd represents the dispersive component, δp the polar and δH the hydrogen bonding. The Hansen parameter solely covers all the molecular interactions in a mole of material, which are dispersion forces, polar interactions (dipole-dipole) and specific interactions such as hydrogen bonding [94]. Using this factor, a good interaction partner for a given polymer should have a solubility parameter close to that of the polymer [94].

CED, on the other hand, is a quantitative measure of how strongly the monomers interact with one another. The Stronger monomers’ interaction, the higher packing densities, and subsequently, the more rigid polymer chains. Chain stiffness hinders molecular movement, reducing the creation of temporary voids for penetration of the molecules to jump into these sites [101]. However, considering MMIPs, this issue is less important because of the thin imprinted polymer layer around the MNP. An ideal imprinting formulation should have an equilibrium of solubility parameters and CED parameters between the monomers and the template. These can be considered as the base of selecting the higher chemical affinity between the template–monomer complexes. Due to the polymerization occurring in solution, the possible effects of the solvents intended to use in the process should be considered as well. Especially in molecular imprinting procedure, the organic solvents are mostly used to greatly affect the physical features of the final complex, including not only its porosity, the surface area and the swelling behavior, but also the pre-polymerization complex stability, which in turn decisively determines the selectivity of the resulting imprinted cavities [102]. It is noteworthy; that the possible residues of these organic solvents in the final complex should be quantified according to the maximum acceptable amount stated by the regulatory authorities in terms of the product safety profile that in the following will be discussed in the related section.

#### 3.1.2. Interaction Mode and Energy Calculation

Calculating the thermodynamic features, namely geometric optimization as well as binding types and energies of each virtual pair of monomer and template becomes a routine computational approach prior to MIP synthesis to predict the most stable complex with the lowest binding energy profile [80,89,90,91,92,93,99,103,104]. Alongside with the atom in molecules analysis of the molecular electron density distribution of the complex to understand the nature of the bonds in deeper detail [89]. However, not all of these studies took the effect of solvents into account [90,91,92,97,99,102,103]. Using ab initio computational methods can bring us with more reliable prediction due to the consideration of the polymerization solvent(s) in the design of MIPs as they can change in energy and stability of the template–monomer complexes [91,97]. In other words, different solvents can cause different template–monomer complexes stabilization energies. This effect can be calculated by the Hartree–Fock method in order to select the most stabilizing system [97]. This method is an ab initio method and shows the relative stability through total energies and relative energies of complexes [89,96].

Herein, to comprehend the best selectivity at the molecular level in pre-polymerization solution with the lowest binding energies, Dramou and coworkers assessed the possible influence of the solvent (DMSO) on the conformation of EPI-loaded MMIPs on the basis of ab initio calculations of the binding energy of the interaction partners in the pre-polymerization. However, ab initio calculation is relatively time-consuming, making it difficult to screen suitable monomers and solvents. MD simulations have been suggested as a fast method to search for optimal imprinting conditions, especially for the screening of functional monomers. This approach is based on classical mechanical force fields that describe non-covalent interactions, Hydrogen bonding, van-der-Waals forces, dipole-dipole, as well as electrostatic interactions [99]. As a result, Dramou et al. used MD simulations for selecting the most suitable monomers in a fast and no reagent-consuming way developed by Li et al. [99]. Employing forcefield parameter Merck molecular forcefield (MMFF94X) [50,104], they determined the mode and energy of interactions between DMSO and monomers as well as template molecule, showing the generation of hydrogen bonds and van-der-Waals interactions and predicted the final conformations using Molecular docking [50]. Piletska et al. also employed MD simulations as the basis of selecting the most energetically favorable structures for the design and development of MMIPs for the controlled delivery of curcumin [95], emphasizing the suitability of this modeling strategy for further studies on the magnetic imprinted systems.

### 3.2. Imprinting Strategy, Advantages of MNPs Surface Imprinting in Drug Delivery

Among the different imprinting approaches such as bulk imprinting, emulsion polymerization, precipitation polymerization, iniferter polymerization, surface imprinting, etc., [62] the favorable grafting of a thin MIP film on the surface of MNPs is possible through surface imprinting or 2D imprinting technique, an easy and straightforward method for fabricating core-shell MIP NPs [49]. This process results in MIPs with imprinted binding sites near to or situated at their surfaces possessing features of both components; that is, MIP high selectivity toward template and high loading capacity with electrochemical and magnetic properties of the core NPs into a single functional hybrid structure (MMIP) [59,62,105] facilitating its distribution and preventing the nanomaterial from being cleared by metabolic burden before reaching the site of action [69]. Therefore, enabling tumor location accumulation via an external magnetic field and vanquishing cancer cells more efficiently with smaller drug dosage and most probably significantly fewer side effects on healthy tissues.

The surface imprinting polymerization exhibits high binding capacities with an exceptional selectivity, especially when compared to other imprinting strategies. The main reason can be mentioned as the high surface-to-volume-ratio of MNP. For example, bulk imprinting, which produces polydisperse particles, irregularly shaped with diffusional limitations of out of access or destroyed potential imprinted sites, generally yields particles with low binding capacities and selectivity. Furthermore, precipitation polymerization in which highly diluted monomer solutions are employed can negatively affect the template–monomer interaction and thus sensitivity and selectivity. Even emulsion imprinting with a potential platform disruption due to the stabilizers/surfactants addition and remaining residual of these additives even after extensive washing steps possess limitations when compared to MIP grafted onto MNPs [62,106]. In these molecular imprinting methods, a high level of crosslinking is used to ensure template binding specificity, and thus the resulting rigid polymeric network hinders the penetration and accessibility of the solvents to the template embedded in this polymer matrix. This extraction does not totally remove the template, which may lead to a suboptimal release rate [104]. On the contrary, however, MMIPs developed by surface imprinting took a step forward by their excellent easily reachable positions of the template due to the thin accessible MIP layer and fast binding kinetics, rapid mass transfer with no or less diffusional problems, reduce permanent entrapment of templates and strong anti-interference ability [62,107].

#### 3.2.1. Optimizations in the Imprinting Process toward Enhancing the MMIPs Physicochemical Features

**Figure 3:**
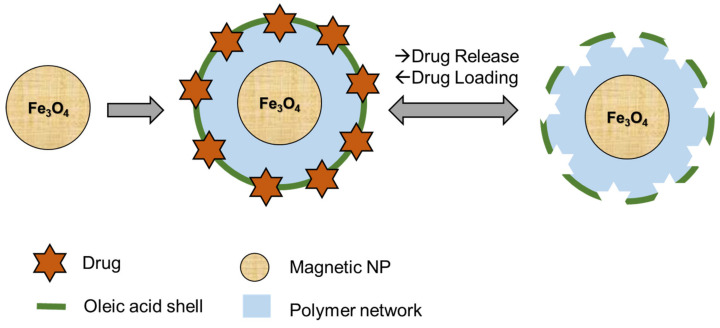
Scheme of the MMIPs preparation with an oleic acid topcoat The pre-polymerization mixture was obtained by the dissolution of the 1.0-mmol epirubicin (EPI) in 10 mL of DMSO and the addition of 9.0 mmol of functional monomer to this solution. The obtained mixture was then added to a suspension of 1.0 g Fe_3_O_4_ nanoparticles (NPs) in 5 mL of DMSO and 20 mmol of ethylene glycol dimethacrylate (EGDMA), followed by adding 100 mL of DMSO–H_2_O (9:1, *v/v*) (containing 0.4 g polyvinylpyrrolidone (PVP)). This pre-polymerization solution was then transferred into a three-necked flask, followed by 0.1 g of 2,2′-azobis(isobutyronitrile) (AIBN). Five hours later, 5 mL of oleic acid was added to the flask. The reaction was kept at 60 °C for 12 h [reworked following [50]] (reprinted with permission of the Royal Society of Chemistry, 2020).

With the aid of the aforementioned computational analyses, Dramou and coworkers selected methacrylic acid (MAA) and methacrylamide (MAM) as the functional monomers, alongside with the EGDMA as the crosslinker and EPI as an anticancer template in the presence of a dispersant (polyvinylpyrrolidone (PVP)) in the mixed media of DMSO–H_2_O illustrated in Figure 3 [50]. The modification of the obtained MMIPs surface was performed using a high amount of oleic acid as a top coat above the imprinted system, giving it an amphiphilic property that makes it water, as well as other solvents, compatible [50,108]. They reported obtaining a good reproduction and repeatability of their designed EPI-loaded MMIPs. We state the loading capacity and release behavior in the related sections. The surface modification of MMIPs with oleic acid is due to this fact that most of the MMIPs are developed in organic solvents, and therefore, they often retain their selectivity in aqueous solvent systems as well as in biologic fluids because of the weaker hydrogen bonding and electrostatic interactions in aqueous media compared to the organic solvents [50,108,109]. Due to the presence of the oleic acid on the surface of the MMIPs, the hydrogen bonding between the template and the polymeric matrix is preserved to water from rapid destruction [108].

Furthermore, Parisi and coworkers reported a developed synthetic strategy based on photo-polymerization with 360-nm light at 4 °C that allows the preparation of magnetic imprinted nanospheres loaded with carbazole derivatives (CAB1, CAB2) at low temperatures, which is essential to avoid any possible drug degradation [3]. The MMIPs were obtained by the precipitation polymerization as followed. The pre-polymerization mixture was formed by the dissolution of 1-mmol of the template and 8 mmol of functional monomer MAA in a mixed solvent including acetonitrile (20 mL)–toluene (20 mL) and followed by the addition of 0.5 g of MNPs in the presence of EGDMA and AIBN and photo-polymerization with 360-nm light at 4 °C for 24 h. Supporting data in the literature also shows that photoinitiated polymerization at low temperatures decreases the kinetic energy of the pre-polymerization complex, which increases its stability and brings more binding capacity and specificity than polymerization with thermal initiation, that mostly requires temperatures higher than 40 °C [38].

Another group reported the preparation of a novel MMIP with dopamine (DA) as the monomer in two parallel studies for the controlled and sustained release of DOX and 5-FU at the tumor site in a breast tumor-induced mouse model [65,79]. This straight forward imprinting process may help future studies to reduce energy in the imprinting process. In this report, imprinting was achieved by dispersion of 0.5 g of MNP in Tris buffer (150 mL, 10 mM and pH 8.5) and the addition of the template following by 0.5 g of DA and 12 h of mechanical stirring, without any external energy source (UV light or heating) at room temperature. The concept is based on the facile self-polymerization of DA to form polydopamine (PDA) coating. PDA’s low toxicity and biocompatibility make it a right candidate for shell materials of MNPs [110]. Alongside the other advantages like lower mass transfer, host for large active groups reactions on the surface and formation of all kinds of material surfaces through covalent and non-covalent interactions [79].

#### 3.2.2. Stimuli-Sensitive MMIPs Triggered Release

As mentioned before, with a proper design, anticancer drugs can be released from their carriers upon a particular stimulation [8,29,35]. The stimuli-sensitivity modulates the affinity of the polymeric network for the template molecule, providing the switching capability of the loading/release processes [37]. With this regard, to achieve a controlled release of the drug out of MMIPs, the advantage of tumor environment chemistry is taken by enhancing the DDSs with a pH/thermo-sensitive trigger [78]. Suitable hyperthermia will directly eradicate tumor cells without damaging nearby healthy cells because of the higher temperature in cancer cells when heating as well as less tolerance of the heat by the cancer cells [71]. A proper magnetic field can cause hyperthermia generated by the MNP core at the vicinity of MMIPs without global heat dissipation, so-called hotspots [68]. Therefore, by a careful selection of thermo-responsive monomers, such as 2-(dimethylamino) ethyl methacrylate, MAA and N-isopropyl acrylamide (NIPAM) [111,112,113], we would have a multifunctional DDS guided and accumulated by the external magnetic field into the tumor location and perishes the cancer cells by magnetic hyperthermia as well as the thermo-triggered release of the anticancer drug from the imprinted polymer [68].

Taking thermo-sensitivity into account, Li and coworkers reported the development of a thermo-sensitive MMIP based on Fe_3_O_4_–carbon NPs and NIPAM as the thermo-responsive monomer for selective adsorption and controlled release of 5-FU from an aqueous solution [80]. Poly-NIPAM (PNIPAM) has a reversible solubility in an aqueous solution at around 32 °C. Therefore, the ability of the resulting MMIP in capturing and releasing template molecules can be adjusted by temperature [111]. Li et al. obtained the multi-core MMIP synthesis in two steps. They first, formed Fe_3_O_4_–carbon NPs (C-MNPs) by the reaction of ferrocene iron (Fe(C_5_H_5_)_2_) in the presence of H_2_O_2_, followed by the silanization of the obtained C-MNPs surface with 3-(trimethoxysilyl)propyl methacrylate (MPS). The second step was the 5-FU–NIPAM imprinting process at the surface of these functionalized MNPs [80]. The logic of the carbon layer around MNPs did not explain by the authors, but it seems carbon film was chosen as the support material, owing to its good acid–base and thermal stability and mechanical stability, as well as rich bonding sites on surface-modified with silane groups [114]. An interesting reversion in the solubility of these MMIPs at around 39.3 °C, compared to the pure PNIPAM (around 32 °C), shows the effect of grafting this polymer on a rigid substrate and the restriction of polymer chains movement, as well as the incorporation of a hydrophobic crosslinker into the system [115]. The PNIPAM shell can swell below this temperature (around 39 °C), leading to access of template to the imprinted cavities and drug loading, as well as the shrink in higher temperatures to become more hydrophobic in an aqueous environment, causing a deformation of the imprinted cavities and drug release. The hydrodynamic diameter of these MMIPs decreased from 282 to 214 nm, as the temperature increases from 20 to 65 °C [80]. Besides the conformation changes of the polymeric network, thermo-triggered drug release out of MMIPs by destabilization and disruption of the hydrogen bonds existing between the drug and the polymer was also studied [68,77] and will be discussed in the section related to the drug release.

Furthermore, almost all tumor tissues have a lower pH (pH = 5.8) compared to the healthy tissues. Hence, pH-responsive polymeric structures have been one of the most prevalent approaches for cancer treatment [116]. pH-responsive polymers are polyelectrolytes with weak acidic or basic groups that either protonate or deprotonate with a change in the pH of their environment [117]. Therefore, the anticancer drug can selectively release from a pH-sensitive polymeric DDS only around the tumor lesion when the environment is acidic [118,119]. The pH responsivity comes from the polymers with ionizable moieties that employs a non-covalent transition to achieve pH responsivity through basic moieties include amines, pyridines, morpholines, piperazines; and acidic groups include carboxylic acids, sulfonic acids, phosphoric acids, boronic acids, which can be protonated or deprotonated at different pH values [116,117]. The (meth) acrylate, (meth)acrylamide, and vinylic polymers are frequently used due to the presence of such groups in their structure [117]. A pH-responsive system based on thermodynamic computational calculations for the preparation of 5-FU-loaded MMIPs was released By Talavat et al. [61]. Following the calculations 4-vinyl pyridine (4-VP) and AA were chosen as optimal monomers to generate pH-sensitive polymers on the surface of vinyl-modified MNPs, prepared briefly by the addition of 0.5 mL functional monomer and 0.5 mg 5-FU to the 100-mL solvent mixture (acetonitrile/methanol (80:20, *v/v*)) and dispersion of 0.3 g of vinyl-modified MNPs, EGDMA and AIBN to this mixture, followed by the polymerization at the reflux temperature of 75 °C for 8 h [61].

As well as Hassanpour and his coworkers that evaluated the pH-sensitivity of MAA and itaconic acid (ITA) as pH-responsive monomers in the preparation of pH-sensitive AZT-loaded MMIPs for use in breast cancer therapy [81]. For this aim, 1 mmol of AZT as the template and 2 mmol of the functional monomer as functional monomer were dissolved in the least volume of acetonitrile and mixed with the dispersion of vinyl-modified MNPs in acetonitrile. Eventually, the polymerization was performed in the presence of EGDMA as crosslinker and AIBN as the initiator at 60 °C for 24 h.

Natural polymers can also display pH-responsive behavior such as gelatin, chitosan, alginate, hyaluronic acid and dextran. Natural polymers have appeal because they display desirable biocompatibility. However, they may not provide sufficient mechanical strength and may contain pathogens or evoke immune/inflammatory responses [120]. For this issue, synthetic pH-responsive polymers were produced from polypeptides such as poly(l-glutamic acid) (PLGA), poly(histidine) (PHIS) and poly(aspartic acid) (PASA). These polymers are biocompatible and degradable like natural polymers [117].

Multiresponsive polymers also have recently utilized a lot to the preparation of polymers that respond to several stimuli, like temperature, pH, biomaterials, redox, light, electrical field, magnetic field, etc. [121,122]. Among these polymers, dual thermo and pH-responsive polymers are the most studied. This class of polymers is prepared by the combination of a thermo-responsive block, such as PNIPAM, poly(N-vinylcaprolactam), poly(N,N-dimethyl acrylamide), with a pH-responsive block, like poly-AA, poly-MAA or poly(N,N-dimethylaminoethyl methacrylate) (PDEEMA) [121]. In general, PNIPAM is chosen as a thermo-responsive block due to its lower critical solution temperature (32 °C) in water that is near the body temperature [117,121].

In this manner, Kaamyabi and coworkers developed a dual pH-thermo-sensitive MMIPs by polymerization of NIPAM on the functionalized Fe_3_O_4_ substrate resulting in multi-core MMIPs for controlled delivery of DOX to the tumor location [78]. The functionalized MNPs (100 mg) was suspended in water–ethanol solution (1:5, *v:v*, 30 mL) followed by the dropwise addition of NIPAAM-DOX complex in water/ethanol (20 mL, 20/80) (prepared by stirring at room temperature for 12 h) and AIBN as an initiator. The polymerization was performed through the overnight stirring of this mixture at 70 °C. A solution EGDMA was added and the reaction mixture allowed to mix up for more six hours [78].

#### 3.2.3. Cyclodextrins as Comonomers

The novel advances in MIT have resulted in the appearance of synthetically engineered MIPs incorporated with cyclodextrins (CDs) in an imprinted polymeric framework with the improved performance [123,124,125]. CDs are cyclic oligosaccharide structures established by d-glucopyranose units consist of 6 (α-CD), 7 (β-CD) and 8 (γ-CD) d-Glucose Monomer, that are linked by glycosidic bonds [126]. The glucose units of CD with a non-twisted chair arrangement conform a narrow half-tapered cavity structure of CD [127]. These highly versatile oligosaccharides owe multifunctional properties that are mostly implemented to elevate the drugs’ solubility, stability, dissolution rate and bioavailability [128].

CDs possess mostly hydrophilic groups on the outer surface and hydrophobic ones on the inner surface inside their cavities. Therefore, hydrophobic drugs can entirely or partially enter within these lipophilic cavities, and a CD-drug complex is formed by host–guest non-covalent bindings. Hence, CDs potentially can improve the problem with the solubility of hydrophobic poorly soluble drugs in aqueous media through encapsulation of such guest compounds into their cavities when they can match with cavities in terms of polarity, size, shape and properties [126,129]. Formation of a stable supermolecular complex between CDs and larger guest structures is also possible through their hydrophobic groups, which can bind into these CDs cavities [126,129]. Taking advantage of this feature, CD derivatives, especially β-CD, has been recently gained interest as a functional monomer at MIT. CD-MIPs are generally composed of CD/derivatives and other functional monomers as binary functional monomers [130].

Most the studies implied CDs into MIPs formulation are developed for in vitro compounds recognition, absorption and separation [131,132]. β-CD- MIPs were successfully employed to recognize, isolate and absorb several biologic compounds, such as peptides, steroids, cholesterols, antibiotics and chemical compounds like pesticides and phthalate [130,133]. CDs were successfully broad-studied in terms of drug delivery. Nevertheless, the combination with MIT to generate a DDS is relatively new. There are only a few studies that investigated the insertion of CDs in MIPs formulation for the aim of drug delivery and release control [48,134,135]. Herein, Sedghi and her coworkers took advantage of this thermo-sensitive MMIP system in combination with acryl functionalized β-CD and curcumin (CUR), as a potential herbal chemotherapy agent, to enhance the CUR solubility, stability, bioactivity and also drug loading and sustained release by β-CDs cavities [135]. The silica-protection of MNPs based on the modified Stöber hydrolysis reaction [136], followed by the surface silanization with MPS provided a suitable base for the imprinting of CUR/vinyl-modified β-CD complex and NIPAM as monomers. A most significant point of their report could be highlighted as the promoted adsorption of CUR into this MMIP system due to the presence of β-CD and host–guest interactions of them [135].

## 4. Results and Discussion

### 4.1. Particles Size and Loading Capacity

When talking about nanoscale in the manner of NPs, they are defined as particles in the nanometer size ranging from 1 to 100 nm [40,49]. However, in the field of molecular imprinting nanoMIPs typically refer to MIPs with diameters up to several hundreds of nanometers [1,137,138]. One of the most important barriers in front of designed MIPs to show their favorable efficacy is the fact that only molecules with a diameter in the range of ≤100 nm can leak from these blood vessels and accumulate within the tumor tissues. Larger NPs show restricted diffusion into the extracellular space, leading limits in their efficacy by preventing them from quickly reaching cancer cells [2].

The recent developed MMIP DDSs are spherical nanomaterials and the average diameter of them can be classified into three general groups: (1) sub-100-nm [65,68,77,78,79], (2) 100–500 nm [61,80,135] and (3) over 500 nm [50,81] (see Table 1). These comparatively large NPs (>100 nm) tend to high accumulation into liver and spleen, resulting in nonspecific clearance by the reticuloendothelial system (RES) and preventing the EPR effect for tumor accumulation, even when an external magnetic field is applied to concentrate the particles in the tumor site. Smaller sub-100-nm NPs like 30 nm micelles have been reported to have the ability to penetrate the poorly permeable tumor, resulting in a higher antitumor efficiency in animal models [139].

Although research evidence on non-imprinted polymeric DDSs has shown improved drug delivery with an increase in efficacy and decreases in side effects, they mostly suffer from low drug-loading capacity and initial fast premature release of the encapsulated drug [140]. It leads to a suboptimal activity at the targeted site and elevated side effects [4], potential toxicity from dose dumping and inconsistent release [2]. The tailor-made affinity between the template and polymer functional groups introduced by MIT leads to a higher loading capacity than the non-imprinted ones. The already published studies reviewed by Bodoki et al. showed the sustained release manner out of MIPs and potential zero-order drug release that could be achieved over long periods. These findings represent a clear advantage over non-imprinted polymeric drug delivery, as they are capable of providing higher loading capacity, more control on drug release behavior and protecting the active ingredient from enzymatic degradation during its transit through the body [4]. Therefore, less dose-dumping toxicity and adverse effects on healthy tissues and more efficacy due to prolonged circulation time are expected [49].

Previously, drug-loading capacity (adsorption) kinetics and isotherms of obtained products have mostly been investigated by the incubation of washed MMIP/MNIP with aqueous template solutions (template rebinding) at different time intervals until the equilibrium (the soaking procedure) and applying the different conditions during this procedure can determine the dose-dependency (the amount of template), pH-dependency, as well as thermo-sensitivity of adsorption pattern [3,50,77,80,135]. The soaking method seems to be favorable for drug-delivery purposes by determining the maximum and optimum loading capacity, considering drug potency and release rate for further in vivo applications. With interest in sub-100-nm MMIPs, The experimental data of Li et al. [80] revealed the equilibrium adsorption capacity (Q) value of MMIPs about 1.5 times higher than that of MNIPs at the steady temperature of 25 °C, suggesting the favorable binding ability of the imprinted system over non-imprinted polymer matrix. The maximum adsorption capacity of MMIPs and MNIPs at 25 °C was reported 96.53 and 59.5 mg/g, respectively. As they elevated the temperature, the Q of MMIPs became lower due to the shrinking of the polymer to become more hydrophobic. It is interesting that, because of the non-specific binding sites in MNIPs, a smaller change in Q value was observed for MNIPs when the temperatures raised [80]. Supporting data were reported by other studies, indicating the higher binding capacity of MMIPs compared to MNIPs due to the presence of the selective imprinted cavities with a high affinity toward the template molecule [50,77,81]. Parisi et al. performed the binding experiments via the incubation of washed MMIPs and MNIPs with a CAB1 standard solution (0.1 mM) for 24 h and reported the loading capacity with the percentages of the bound CAB1 by imprinted and non-imprinted nanospheres that were 52% and 38%, respectively [3].

To study the effect of pH on the drug-loading capacity of MMIPs, Hassanpour et al. incubated the particles with AZT solutions (25 ppm) with a pH range of 3 to 11 for 4 h. Their results indicated that the adsorption percentage of AZT on MMIP reached to its maximum value (60%) at pH 5 (related to the protonation of AZT functional groups and deprotonation of the carboxyl groups of the imprinted cavities, respectively) and then slightly decreased by a further increase of pH. In contrast, MNIPs showed much lower loading capacity of around 19% at pH 5 and smaller capacity in other pH s [81]. Dramou et al. also reported the same pH-dependent adsorption behavior of EPI into the MMIPs in two directions and the existence of a climax pH point 5.8 [50], which may contribute to the counteraction between the decrease and the increase of the hydrogen bonding interaction between the template and polymeric matrix [50,81].

### 4.2. Toxicity and Degradability Studies

Although the development of nanocarriers intended for drug delivery has become more robust, there are still some critical knowledge gaps in terms of their safety profile. The safety of a complex depends on the safety of each material involved and the unpredictable effects when acting as one unit [4]. NPs can cause toxicity and immunogenicity due to the relative size, shape, chemical composition and surface charge. Vital organs like the spleen accumulate particles larger than 100 nm, while the pores in the liver are about 100 nm, and can cause the aggregation of the smaller materials [7]. As a result, the number of nano-DDSs approved for chemotherapy is scant, and they are mainly based on liposomes, which are biocompatible and biodegradable and with a bilayer structure analogous to that of the cell membrane [4].

MIPs were reported in recent years more for the application in the delivery and controlled release of anticancer agents [25,35,54,61,65,66,96,141,142,143]. However, most of the studied MIPS supposed for demonstrating their relevancy as DDS use formulations (type and molar ratios of functional monomers, crosslinkers and solvents) ab initio tested for analytical applications [144] and a few in vivo animal model assessment of the MIP-DDS were performed [4]. Most of the presently developed MIPs are nonbiodegradable, which may be dangerous due to their bioaccumulation in blood vessels or cells, tissues and organs after administration [49].

Biocompatible materials are biologically compatible without making local or systemic reactions of a living system or tissue [145]. The materials of the intended MMIPs for drug-delivery purposes need to be selected very carefully due to the most critical issue, which is nontoxicity, biocompatibility and biodegradability. The biocompatibility of NPs is a vital aspect of their medical applications. Thus, NPs should integrate with biologic systems without immune response stimulation and any toxic accumulation [146].

As an example, Asadi and coworkers introduced a multi core-shell MMIP loaded with 5-FU based on biodegradable materials for targeted, sustained and controlled release of the drug in vitro analyses onto Michigan Cancer Foundation-7 (MCF7) cells. Herein, tannic acid as a biodegradable polyphenol was used to fabricate the crosslinker [54]. Tannic acid is a natural crosslinker owing to the presence of hydroxyl and carboxyl groups that can interact with biopolymers. The biodegradable structure of the obtained imprinted system was shown, using various conditions similar to the body. Data revealed that, due to the acidic nature of this crosslinker, the degradation of particles in higher pH, similar to the kidney and intestine environment is faster.

The liver and the spleen catch most NPs in the bloodstream as organs of the mononuclear phagocytic system (MPS). It is noted that iron oxide NPs are captured within the MPS via endocytosis into Kupffer cells of the liver sinusoid and macrophages of the spleeny red pulp, where they undergo degradation within the lysosomes of these cells. The degraded iron is finally eliminated from or restored in the body via normal iron metabolic pathways with no or low signs of in vivo hematotoxicity and blood chemistry effects [147,148]. Indeed, biocompatibility and degradation of iron oxide NPs have been well monitored in the past few years [144,147,148,149], but only a small number of papers deal with the toxicity of imprinted polymers [64,144,150,151,152].

Recently, biodegradable MIPs have been developed by using biodegradable crosslinkers or monomers in the imprinting systems [52,53,64,137]. Both (semi) synthetic and biopolymers are available for use in NP DDS. Typical functional monomers used in the imprinting process are carboxylic acids (AA, MAA, vinylbenzoic acid), sulfonic acids (2-acrylamide-2-methylpropane sulfonic acid), heteroaromatic bases (vinylpyridine, vinyl imidazole) [57]. However, biopolymers such as chitosan, albumin, alginate, dextran or collagen have gained many attractions for pharmaceutical applications as they are inexpensive, biocompatible, chemically modifiable, biodegradable and allow simple control of the size and surface properties of the resulting system [153]. Chitosan is an excellent candidate among biopolymers, which has remarkable properties and the presence of functional groups. It is widely used in the encapsulation or coating of various types of NPs [154] and as functional [155] and supporting matrix [156] in the imprinting process of MIPs and MMIPs, showing strong potential in many fields like curbing environmental pollution, protein separation and identification, chiral-compound separation and medicine [157,158,159]. However, concerns associated with their relatively fast release profile, purity and homogeneity and more important, the toxicity of natural compounds present a greater challenge of using them as medicine [4,32,154]. Consequently, many natural NPs are not clearing the clinical trial phases [154].

Herein, the monomers implemented in the preparation of the MMIPs for drug delivery can be subdivided into two general categories: natural elements like DA and oleic acid, as well as (semi) synthetic monomers whether vinyl monomers including AA, MAA, MPS, PVP, 4-VP or acrylamide (AAM) derivatives like NIPAM and methacrylamide (MAM). Generally, polymers with hydrolyzable backbones like vinyl polymers containing easily oxidizable functional groups are susceptible to hydrolysis and enzymatic biodegradation [160]. The ability of artificial or natural biodegradable polymers to be cleaved into biocompatible byproducts through chemical or enzyme-catalyzed hydrolysis make it feasible to optimize the safe removing of these structures in the body [54]. Polyacrylamide (PAAM) is widely used in biomedical applications and itself is not significantly toxic [161]. However, neurotoxicity, reproductive toxicity and carcinogenicity of its monomer, AAM, in animal species have been documented [162,163]. AAM monomer residues are probably an impurity in most PAAM preparations, ranging from <1 ppm to 600 ppm. Higher levels of AAM monomers are present in the solid form of the polymer [161]. Darnell and coworkers made a study to identify the biocompatibility of extremely tough alginate/ PAAM hydrogels. They reported a statistically significant reduction in cell metabolic activity with PAAM gels suggesting latent AAM monomer as a potential source of such reductions. However, following histology of the tissue surrounding the gels showed an absence of immune cells, suggests that in vivo exposure to latent AAM monomer is minimal. Therefore, they concluded minimal effects of this compound on cells in vitro and in vivo, [163]. Use of this polymer and its derivatives in foods, drugs and devices is regulated by the FDA, with restrictions on the amount of PAAM that can be used, and the AAM residue in either the polymer or in the final product is restricted and should be monitored closely [161].

The most studied thermo-responsive polymer used in the preparation of thermo-sensitive MIPs and MMIPs is the PNIPAM, but its potential toxicity emphasizes close monitoring and using alternatives when possible. Studies demonstrated excellent thermo-sensitivity of the polymers based on oligo(ethylene glycol) methacrylates (OEGMAs and in vitro or in vivo biocompatibility already validated by the FDA [164,165]. Sousa-Herves et al. developed a multi-responsive DOX-loaded nanogels focusing on pH and redox-sensitivity by using monomethyl oligo(ethylene glycol) acrylate (OEGA) and pH-responsive 2-(5,5-dimethyl-1,3-dioxan-2-yloxy)ethyl acrylate (DMDEA) as monomers and Redox-responsive bis(2-methacryloyl)oxyethyldisulfide (BMADS) as the crosslinker. They reported that approximately 95% of DOX was released after 8 h at pH 5 in the presence of a reductive agent, while only 20% of the free drug could be observed for the same incubation time at pH 8 [166]. In addition, Cazares-Cortes et al. prepared dual pH-thermo-responsive magnetic nanogels, based on OEGMAs and MAA comonomer and demonstrated the desirable thermo-sensitivity of the obtained nanogels to the magnetic-induced hyperthermia [68,167]. Under AMF, the MNPs inside nanogels act as nanoscaled hot spots. This heat was sufficient to cause the shrinkage of the nanogels and the drug release subsequently (32% DOX release after four hours at 50 °C), while the negligible release was observed when these nanogels solutions were stored at 4 °C (less than 20% after one month) [167].

Moreover, potentially, photopolymers are known to release residual monomers, photoinitiators and similar products resulting from decomposition processes into the environment [168] with the logic that when the water permeates into the matrix, the leachable unreacted monomers diffuse out [145]. Although hydrophilic monomers were identified in higher proportions in aqueous extraction media than hydrophobic once [169], it is accepted that some unreacted methacrylate groups are not capable of being leached into aqueous media due to their covalent bounds to one end of the polymer chain [145]. The imprinted polymer layer is relatively thin as grafted onto the magnetic core, and the chance of remaining unreacted monomers in the polymer matrix is expected to be little.

Furthermore, most the imprinted systems are developed and evaluated in non-polar organic solvents such as toluene, chloroform, dichloromethane or acetonitrile as they depend on potent hydrogen bonding and electrostatic interactions formed in these organic solvents [38,50]. The solvent brings all the interaction partners into one phase in the polymerization and creates pores in the polymer matrix for further access to the embedded templates in the imprinted system. However, the presence of these organic solvents may cause cellular damages. Therefore, in drug-delivery processes, it is vital to prepare MIPs in such a way that they are compatible with biological systems [38]. One of the issues that face MMIPs is their lower selectivity when tested in aqueous solvent systems. MIPs usually perform optimal recognition of the template in the same solvent as the one used in their preparation process [50]. This can be due to the considerable weakness of hydrogen bonding and electrostatic interactions in water. However, people are working to develop strategies to make the imprinting process efficient enough in polar media. There are reports regarding the preparation of efficient MIPs in rather polar solvents (e.g., acetonitrile/water, ethanol or methanol/water) since strong template–monomer interactions have been observed [78,107,170]. Metal coordination and hydrophobic interactions are also suggested to enhance template and functional monomer interactions [171,172]. In addition, one of the advantages of MMIPs is the thin imprinted polymeric matrix around the MNPs, leading to the decoration of the imprinted cavities almost at or near the surface. Therefore, due to the less penetration needed for the extraction of the template, the possibility of using less porogenic solvents exists for the imprinting process.

### 4.3. In-Vitro Drug Release Behavior and Cytotoxicity, In-Vivo Experimental Studies

From DDS aspects, the first step of almost every NP in vitro evaluation is to determine the release behavior, which is carried out mostly using the dissolution methods and sampling during the specific periods [3,50,95]. Although studies analyzed the effect of different temperatures and pH on the drug release behavior, several further parameters are affecting the release rate and behavior of a drug from its carrier in vivo. To name a few, tissue homeostasis, protein corona around the particle (the unspecific adsorption of proteins on the MMIP surface that must be avoided because it could interfere with the interactions with the template [3]) and immune response (that can be prevented by using biocompatible materials). Therefore, if the dissolution platform resembles the biologic environment as much as possible the release behavior in vitro is expected to be near to the biologic condition, suggesting phosphate-buffered saline or simulated body fluid (SBF), which is similar to the human body plasma and has an ionic composition, pH 7.4 at 37 °C ± 0.5 °C [61,65,78]. Due to the acidic environment of cancer cells (pH 5.8), it is worthy of investigating the release behavior in acidic conditions [78]. However, in general, high physical adsorption on the surface and the nonspecific bonds cause a rapid release in the initial phase to be observed in most release process curves [3,65,79,135], followed by a sustained release process that seems to go on for a prolonged period, related to the drug release from imprinted binding sites. To avoid complications on other healthy organs, the MMIP can be directed by an externally placed magnet to the target tumor site in the body, thanks to the presence of magnetic core and the compatibility with an aqueous environment [50].

The in vitro release profile of the CAB1 from MMIPs and MNIPs in SBF at 37 °C was reported as following [3]. About 19% of the total loaded CAB1 was released from the imprinted matrix during the first hour, while MNIPs released about 49% within this period. The MNIPs released the drug completely within 6 h, while in the case of MMIPs even after 48 h the drug release was not complete. It shows a sustained release upon imprinted polymers that can reduce the massive adverse effects of off-target release toxicities.

To demonstrate the effect of the alternating magnetic field (AMF) on the drug release profile, Griffete group compared the release rate from thermo-sensitive DOX MMIPs placed under AMF with the same particles that were left at 37 °C for the same period. The release rate was reported by 60% and 10–15%, respectively. The authors suggested the thermo-triggered drug release out of MMIPs is performed by destabilization and disruption of the hydrogen bonds existing between the drug and the polymer. They also reported a high drug release from thermo-sensitive DOX-loaded MNIPs that were subjected to the same condition as imprinted particles. The high release rate from MNIPs placed under AMF compared to those left at 37 °C was described as 73% and 98%, respectively, showing AMF-unspecific, non-sustained drug release patterns compared to MMIPs. Taking together, these results bold the significance of the magnetic core as nano hot spots that trigger the release of the drug out of the cavities only upon a suitable AMF [77]. Furthermore, Cazares-Cortes et al. compared the release behavior of DOX out on MMIPs with non-imprinted magnetic nanogels (MagNanoGels) that physically encapsulated the drug. According to their results, the release amount of DOX from the nanogels was two-fold more than MMIPs under the AMF (16.7 µM compared to 7 µM), but in terms of percentage, the MMIPs released more than half of the DOX trapped in their matrix. Additionally, MMIPs demonstrated a low passive release pattern (10%) without AMF at 37 °C and pH 7.5, compared to 24% for MagNanoGels in similar conditions, emphasizing the effect of imprinting on the better control over the drug release on-demand [68].

Followed by the design of a dual-pH-thermo-responsive DOX-loaded MMIP explained before, the release profile was investigated in the SBF at different pH s. The drug release rate elevated significantly by increasing the temperature from 37 to 40 °C. They reported a huge difference between the release rate in pH 7.4 and pH 5.8 after 144 h (12% compared to 70%, respectively). DOX was almost released in the cancer cell’s acidic pH, but only 12% are released in the pH related to healthy tissues. Therefore, the use of this imprinted system is promising to deliver more DOX to the carcinogen cells and reduces damage to healthy cells. This release pattern could be attributed to the fact that the hydrogen bonds, which cause DOX-loading, become weak in acidic pH s and this leads to a faster release [78]. However, to show the thermo-responsivity of the developed MMIPs, the reports of Li et al. [80] from their in vitro drug release experiments of their designed 5-FU-loaded MMIPs are stated here. It was seen that the release amount and release rate for MNIPs are much higher than those of MMIPs at 25 °C within 100 min. Nearly 70% of loaded 5-FU by MMIPs is released, whereas 84% of loaded 5-FU by MNIPs is released at 25 °C, which attributes to the more specific adsorption of the drug in imprinted cavities. They reported the elevated drug release amount (90.75%) from MMIPs when the temperature rises to 45 °C due to the shrinkage of the polymer matrix to become hydrophobic. The release amount from MNIPs was not demonstrated by the study [80].

Cytotoxicity studies against cancer cells give more specific biologic perspectives into the aim of a drug-loaded MMIP intended to use as cytotoxic agents. Parisi and coworkers conducted their developed MMIPs on HeLa and MCF-7 cancer cells to investigate the potential application of the magnetic imprinted nanospheres as a drug carrier in targeted cancer therapy. They reported sharp retardation in HeLa cells growth after 2 and 3 days of treatment with MMIP–CAB1, compared to the relative control (DMSO). The effect was even more evident in MCF-7 cells, which, concerning DMSO treated cells, showed dramatic growth retardation already after one day of MMIP–CAB1 treatment, reaching an almost complete growth inhibition on day 3 [3].

Drug release tested in the PC-3 cancer cell line was investigated and MMIP NPs were efficiently captured by cancer cells [77]. The results demonstrated explicit drug intracellular localization, captured inside intracellular endosome-like compartments. Surprisingly, this MMIP internalization did not induce cancer cell death, with this explanation that when bonded to the MIP (and thus the NPs), the drug is inactive. By contrast, after the athermal AMF application (steady temperature of cell environment at 37 °C), cancer cell viability was affected, with cell viability reduced to 60% after a 1.30-h treatment, corresponding to the cell cytotoxicity rate with free drug after 2 h incubation. These cellular experiments support the AMF-induced drug release and demonstrate the possibility of initiating chemotherapy via an athermal magnetic hyperthermia strategy through nano hot spots by magnetic cores. This remote magnetic activation is particularly promising to limit the adverse effects of chemotherapy on bystander tissues [77]. Similar results were reported of prepared DOX MMIPs with the same study design [68].

Hassanpour et al. demonstrated pH related cell-based studies with three factors: cell viability, cell toxicity and caspase-3 assay percentage of free AZT, AZT-loaded MIPs and MMIPs on MCF-7 and MCF-10 cancer cell lines. The results showed that due to the acidic cancer intracellular medium (pH 5), the drug was released from its imprinted carrier only inside these cancer cells. Nevertheless, in normal healthy cells or blood circulatory system (pH 7.4), carriers released only about 14% of their loaded drug. This ability could notably elevate the drug concentration in cancer cells and subsequently decrease the dose-dependent side effects. They reported that induced cell cytotoxicity in MCF-7 for MMIP, MIP and free AZT was 91%, 71% and 11%, respectively. Cell cytotoxicity induced by MMIP was about 49 times more than free AZT [81]. These findings clearly show the likely controllable and effective role of MMIPs as smart carriers in chemotherapy.

The development of MMIP NPs as a DDS for cancer treatment is in its early stage of development. Therefore, the number of studies performing in vivo experiments with the aim of chemotherapy applying MMIPs is still seldom. Hashemi-Moghaddam et al. developed the study of their 5-FU-loaded MMIP NPs on female tumor-bearing BALb/C mice with a tumor diameter of 80 mm to 100 mm generated of murine mammary adenocarcinoma cells (MMAC; derived from M05 cell line) and compared treatment with free 5- FU, 5-FU-imprinted polymer (IP) and 5-FU–IP in the presence of a magnetic field. Alongside with the evaluation of drug distribution by analyzing the concentrations of free 5-FU or 5-FU–IP in the tumor, liver and kidney tissues through high-performance liquid chromatography (HPLC) to address the potential side effects related to the systemic distribution of each treatment. The HPLC results in these tissues show a high 5-FU–IP accumulation into the kidney due to being close to the magnetic source, but still, show the considerable accumulation of the DDS inside the tumor compared to the liver when there is no significant difference between the amount of free drug accumulation in different tissues [65].

**Figure 4:**
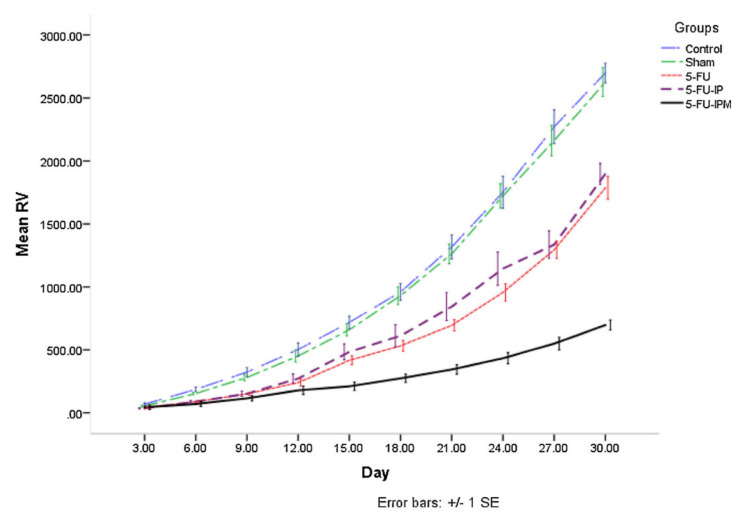
Tumor growth curves of tumor-bearing mice that received different treatments. RV—relative volume; Sham—non-implanted MNP; 5-FU—5-fluorouracil; 5-FU–IP—5-fluorouracil imprinted polymer; 5-FU–IPM—5-fluorouracil imprinted polymer with a magnetic field [65] (reprinted with permission of Elsevier, 2020).

Critical physical parameters to indicate the treatment effectiveness was the relative tumor volume percentage (Figure 4), that was lower in the group exposed with 5-FU–IP in the presence of the magnetic field, resulting in higher control of tumor growth than treatment with free 5-FU and an increase in animal life span. Indeed, by targeting 5-FU to the tumor site and enhancing its local uptake via a magnetic field, unwanted side effects are reduced, and the therapeutic efficiency is enhanced. Indeed, an increase of tumor growth inhibition ratio (IR%) between the 6th and 30th day after treatment in the 5-FU–IP-treated group with the magnetic field was reported 47% to 71%, respectively. In the free 5-FU-treated group, these values were reported from 15% to 48% between the third and twelfth day and thereafter began to decrease, indicating a transient control of tumor growth in this group [65]. Very similar outcomes were reported by the same group for the DOX-loaded MMIP NPs with the same experimental design [79].

## 5. Prospects and Conclusions

As reviewed above, MMIPs are gaining more interest in the preparation of an efficient targeted DDS especially for cancer treatment and it can be predicted that more imprinted magnetic assisted DDSs will emerge. MMIPs are suitable for in vivo applications due to their superparamagnetic properties, which allows them to show no magnetization after removal of the magnetic field [59,173]. Their capability to be guided and induce hyperthermia with an appropriate external magnetic field introduces promising ways for cancer treatment, using the MMIPs as smart-drug-delivery robots, a potential alternative to conventional, systemic direct administered chemotherapy [59]. To date, these systems are generally investigated in terms of their preparation method stabilization, physicochemical properties, selectivity toward templates, loading capacity, in vitro cytotoxicity and comparatively simple in vivo tests. The more important issues regarding their safety and side effects, such as the specific interaction of these systems with human organs, tissues, cells or biomolecules, the effect on human’s metabolism brought by the MMIPs, and the wider application of these systems for drug delivery, await further deep study, which should be focused on shortly.

The basis for the creation of a selective strong molecular imprinting of MIPs lies in the formation of stable template-functional monomer adducts in the pre-polymerization reaction mixture. Hence, the choice of functional monomers to form such stable complexes with the template is vital [98]. Computational modeling proved itself as a powerful tool for the rational selection of the functional monomers and design of the MMIPs prior to the experiment to prevent waste of time and resources as well as increase the imprinting efficiency. MD simulations have been suggested as a fast method to search for optimal imprinting conditions, especially for the screening of functional monomers.

Nowadays the demand for the development of safe body compatible and degradable DDSs forces the use of such materials in the preparation of delivery systems. As a result, the use of biopolymers like chitosan in the preparation of imprinted systems is getting attention and suggesting further studies to solve the related issues and achieve a controllable efficient system for drug delivery in cancer therapy.

Most published studies have implemented thermally initiated free-radical polymerization in order to synthesize MIPs and MMIPs [61,107,174]. However, photoinitiated free radical polymerization offers several advantages different from thermally initiated free radical polymerization [175]. Photoinitiated polymerization is a green method that allows the polymerization to be carried out under more mild conditions, under air, upon blue light exposure, under low light intensity, no need to heat the system and low pressure used. In addition, due to the easiness of turning the light on or off, spatial and temporal control of the initiation step can be reached; such behavior is not possible by heating. Thus, the development of new initiating systems able to initiate polymerization in such conditions is at the center of numerous research [176] due to the low-temperature conditions, facilitation of temporal and spatial control—and especially for practical application solvent-free formulation wavelength flexibility and high curing speed [175]. Nevertheless, as mentioned before, photopolymers possibly release residual monomers, photoinitiators and similar products resulting from decomposition processes into the environment [168]. One of the most studied toxic products is tetramethyl succinonitrile (TMSN) that is released from AIBN as the main product of its decomposition and is built into the polymerized plastic product. Animal experiments in rodents have revealed that TMSN could act as a potent convulsant, which leads to the death of animals due to asphyxia [177]. Therefore, the choice of suitable initiator and close monitoring of its residues in the final product as given in the Code of Federal Regulations (CFR), FDA or other relative regulations is crucial.

To date, the advances in MIT have led to the development of novel synthetically engineered MIPs material that is incorporated with α/β/γ-CDs within an imprinted polymeric framework, which fortunately improved the performance of MIPs [123,124,125] due to their hydrophilic outside and hydrophobic inner space and formation of a non-covalent complex with the guest molecule. Hydrophobic drugs can enter the lipophilic CD cavity with their whole structure or partially. CDs were successfully broad-studied in terms of drug delivery as the functional monomer combined with other functional monomers as binary functional monomers [72]. Even the combination of CDs with MIT to generate a DDS is relatively new [49,134,135], it is a highly promising step forward. In one of the reviewed papers, the prepared MMIP containing β-CD as a monomer showed imprinting ability of this material on the surface of MNPs, high affinity and high adsorption capacity of the imprinted film toward the template and adsorption equilibrium in a short time. These findings are showing the opportunity of further studies with the combination of CDs and MNPs for DDS [135].

Furthermore, most NPs in the systemic circulation are recognized by RES and get accumulated in the liver and spleen leading to toxicity to other organs [28], suggesting the need for utilizing a stealth approach to overcome this biologic barrier. Poly(ethylene glycol) (PEG) is an FDA approved polymer that has become the most widely used “stealth” polymer in drug delivery. Due to its flexible and hydrophilic nature, on NP surfaces it can form a dynamic hydration barrier, which prevents the plasma proteins binding (also termed opsonization) on the surface of the particles and clearance by the MPS, respectively, [178] suggesting the use of such an approach on the surface of future MMIPs intended for drug delivery.

Although there is still a great need for further studies about the application of anticancer agents- loaded MMIPs toward cancer treatment, the here represented reports so far showed promising potential in developing an effective system with high drug loading and controllable guidance with AMF to the tumor local. A desirable controlled drug release with pH-thermo-sensitive triggers, as well as considerable cytotoxicity toward cancer cells compared to the free drugs and a good ability of tumor growth retarding was reported by these studies. All together highlights the need for further detailed studies to develop biocompatible and biodegradable imprinted systems as DDSs for in vivo investigation related to the specific interaction of these systems with animals and human organs, tissues, cells or biomolecules and those possible effects on human’s metabolism. However, first, the existing important limitations such as their behavior in the aqueous media, binding kinetics and slow leaching of the template from the polymeric matrix, need to be addressed for the application of MMIPs.

## Figures and Tables

**Table 1:** Overview of functional monomers and magnetic molecularly imprinted polymers (MMIPs) approximate size.

Polymerization Reaction	Monomer(s)	NP Size [nm]	Imprinted Polymer Thickness [nm]	Ref.
Thermal free radical	AA, MAA, MAM, 4-VP	500	n.m.	[50]
Thermal free radical	AA, AAM	57	26–31	[68,77]
Self-polymerization	DA	80–100	n.m.	[65,79]
Low temp. Photo-pol.	MAA	n.m.	n.m.	[3]
Thermal free radical	NIPAM	80	40	[78]
Thermal free radical	NIPAM	152	50	[80]
Thermal free radical	NIPAM, β-CD	130–150	90–110	[135]
Thermal free radical	AA, 4-VP	200–400	n.m.	[61]
Thermal free radical	MAA, ITA	≥800	n.m.	[81]

n.m.—not mentioned; AA—acrylic acid; MAA—methacrylic acid; MAM—methacrylamide; 4-VP—4-vinyl pyridine; AAM—acrylamide; DA—dopamine; NIPAM—*N*-isopropyl acrylamide; β-CD—β-cyclodextrin; ITA—itaconic acid.
